# Validated risk prediction models for outcomes of acute kidney injury: a systematic review

**DOI:** 10.1186/s12882-023-03150-0

**Published:** 2023-05-09

**Authors:** Fateme Nateghi Haredasht, Laban Vanhoutte, Celine Vens, Hans Pottel, Liesbeth Viaene, Wouter De Corte

**Affiliations:** 1 Department of Public Health and Primary Care, KU Leuven, Campus KULAK, Etienne Sabbelaan 53, Kortrijk, 8500 Belgium; 2 ITEC - imec and KU Leuven, Etienne Sabbelaan 51, Kortrijk, 8500 Belgium; 3 Department of Nephrology, AZ Groeninge Hospital, President Kennedylaan 4, Kortrijk, 8500 Belgium; 4 Department of Anesthesiology and Intensive Care Medicine, AZ Groeninge Hospital, President Kennedylaan 4, Kortrijk, 8500 Belgium

**Keywords:** Acute kidney injury, Chronic kidney disease, Poor renal outcomes, Machine learning, Prediction model, Systematic review

## Abstract

**Background:**

Acute Kidney Injury (AKI) is frequently seen in hospitalized and critically ill patients. Studies have shown that AKI is a risk factor for the development of acute kidney disease (AKD), chronic kidney disease (CKD), and mortality.

**Methods:**

A systematic review is performed on validated risk prediction models for developing poor renal outcomes after AKI scenarios. Medline, EMBASE, Cochrane, and Web of Science were searched for articles that developed or validated a prediction model. Moreover, studies that report prediction models for recovery after AKI also have been included. This review was registered with PROSPERO (CRD42022303197).

**Result:**

We screened 25,812 potentially relevant abstracts. Among the 149 remaining articles in the first selection, eight met the inclusion criteria. All of the included models developed more than one prediction model with different variables. The models included between 3 and 28 independent variables and c-statistics ranged from 0.55 to 1.

**Conclusion:**

Few validated risk prediction models targeting the development of renal insufficiency after experiencing AKI have been developed, most of which are based on simple statistical or machine learning models. While some of these models have been externally validated, none of these models are available in a way that can be used or evaluated in a clinical setting.

## Introduction

Acute kidney injury (AKI) among hospitalized patients is characterized by a sudden decline in renal function and is associated with poor long-term and short-term outcomes [1]. The overall incidence of AKI in hospital patients ranges between 7 and 22%, and it ranges from 20 to 50% in Intensive Care Unit (ICU) patients [2, 3]. Increasing incidences of AKI have been reported, especially among low- to middle-income countries [4], and this is likely due to an increasingly complex patient population. Moreover, it has been shown that when sepsis is present at ICU admission, the prevalence of AKI is greater than 40% [5].

The definition of AKI has changed over the years. In 2012, the Kidney Disease: Improving Global Outcomes (KDIGO) unified the previous definitions (RIFLE and AKIN) [6]. By KDIGO definition, AKI is diagnosed by an absolute increase in SCr, at least 0.3 mg/dL (26.5µmol/L) within 48 h or by a 50% increase in SCr from baseline within 7 days, or a urine volume of less than 0.5mL/kg/h for at least 6 h. Although KDIGO is now the most accepted and used AKI criteria, recently Sparrow et al. [7] evaluated the impact of further sub-categorizing the KDIGO-defined AKI stage 1 into two stages based on SCr criteria: stage 1a (an absolute increase of SCr of 0.3 mg/dL within 48 h) and stage 1b (a 50% relative increase in SCr within 7 days) and therefore creating a 4-stage KDIGO classification which they named KDIGO-4. In a separate study, Nateghi Haredasht et al. [8] showed that within the KDIGO AKI stage 1, there are indeed two sub-populations with different clinical outcomes.

Traditionally, two functional biomarkers, serum creatinine (SCr) and urine output have been used to diagnose AKI. The sensitivity and specificity of these biomarkers are limited, however, due to delayed changes following kidney injury. Cystatin C (CysC), another kidney biomarker, has gained a great deal of attention in the past few years for its use in calculating GFR. There have been multiple studies that show that CysC is a more reliable indicator of kidney function than SCr [9–11]. In addition to Cystatin C, NGAL has also gained significant attention as a reliable biomarker for the early detection and diagnosis of AKI. NGAL can detect kidney injury much earlier than SCr and urine output, which can delay the diagnosis of AKI. NGAL has also shown good correlation with AKI severity and can predict the risk of AKI progression and poor outcomes. Therefore, NGAL is considered a valuable tool for improving AKI diagnosis and treatment [12–14].

AKI contributes to adverse short-term and long-term outcomes. Different studies have linked AKI to the development of acute kidney disease (AKD), chronic kidney disease (CKD), end-stage kidney disease, longer hospitalization time, cardiovascular disease (CVD), and other complications, suggesting that even a short episode of acute kidney injury might lead to long term morbidity [15] and mortality [16, 17]. Among the 19,249 hospitalizations included in a study in which the incidence of AKI was 22.7%, Wang et al. [2] reported the mortality rate was 10.8%, compared to 1.5% for cases without AKI. Moreover, it has been reported that critically ill patients with dialysis-requiring AKI experience mortality rates above 50% [18]. The mortality rate of this sudden kidney failure in ICU is approximately 30–50% depending on the medical record of the patient and the stage of AKI [19, 20].

**Fig. 1:**
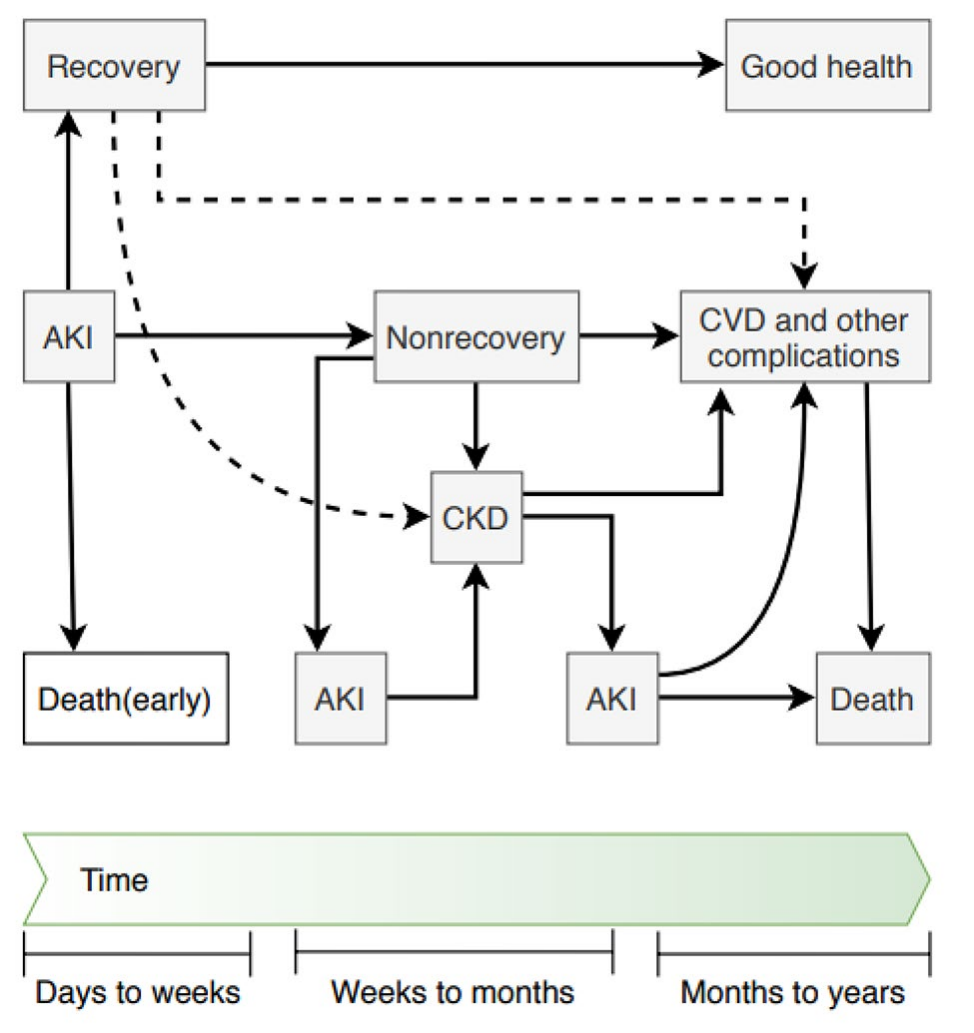
*Possible outcomes following AKI. As a result of an episode of AKI, patients may recover, be discharged without recovery of renal function, or die. Patients who seem to recover may also later develop CKD or CVD (dashed lines)- modified from reference* [27]

Traditionally, most studies of severe AKI have concentrated on short-term outcomes often evaluated at hospital discharge. However, AKI may exhibit important independent effects on the outcome that may extend well beyond discharge from the hospital [21]. Figure 1 shows the potential long-term outcomes of AKI. As a result of an episode of AKI, patients may recover, be discharged without recovery of renal function, or die. Patients who seem to recover may also later develop CKD or CVD.

In recent years, it has become clear that AKI is not a completely reversible syndrome. It is possible that the injury that occurs may result in permanent kidney damage (e.g., CKD) and even damage to other organs. This caused a shift from AKI being a life-threatening and acute situation to a situation with a larger population in need of chronic follow-up to prevent further deterioration of their kidney function [22].

While AKI and CKD have been associated, confounding factors and bias can explain this, thus questioning their causal significance [23]. Nevertheless, in light of the association and the increasing number of patients with AKI (so-called AKI survivors), and CKD, the prediction of CKD after an AKI episode has become increasingly crucial in order to allocate the necessary amount of follow-up to the right patients.

Currently, follow-up of AKI survivors is often lacking and not regulated [24]: follow-up of kidney function by a nephrologist in patients surviving an episode of AKI treated with renal replacement therapy (RRT) is stated in nearly one-third of the patients [26]. Close follow-up and interventions aimed at preserving kidney function may positively impact long-term outcomes as major adverse kidney events have been reported that are common in AKI survivors [23]. However, this is costly and time-consuming. As a result, instead of monitoring all the patients experiencing AKI, it would be useful to identify those subgroups of patients who are at higher risk of developing CKD and only follow up with those patients. In order to do so, we need to collect data to be able to develop a prediction model to output a risk score for developing CKD for patients who experienced AKI.

Lately, with the help of technology, e.g., electronic health records (EHR), collecting clinical and biochemical data is much more straightforward than before [28]. As a result, the resulting data could be analyzed, and prediction models could be constructed. Recently, there have been several studies using machine learning technology for outcome prediction using EHR data [29, 30]. One of the main tasks considered in machine learning is the development of a model by learning from a set of observed data in order to predict outcomes or events for future data [31]. Although the traditional statistical approaches appear to be more appropriate when a large number of cases exceed the number of variables under study and significant a priori knowledge of the subject area is available, machine learning algorithms can handle a large amount of data with high-dimensional variables. In addition, interpretable machine learning models make it possible for healthcare experts to make individualized decisions that will eventually lead to a higher standard of care.

## Objective

In this systematic review with meta-synthesis, we investigate the use of validated predictive models (machine learning or statistical models) for predicting the development of renal insufficiency in the short-term and long-term after AKI scenarios in the hospital/ICU. The term renal insufficiency describes poor kidney function and CKD is the permanent and progressive state of renal insufficiency.

Since it is essential to assess the degree to which a model generalizes, we focused specifically on models that have been validated either externally (e.g., separate cohort) or internally (e.g., cross-validation). Validating a prediction model plays a particularly important role in the healthcare domain since the ultimate purpose of developing a model is to use it in clinical settings, and providing a validated mode enhances its reliability.

## Materials and methods

Published guidance (CHARMS, TRIPOD, and Preferred Reporting Items for Systematic Review and Meta-Analysis (PRISMA)) helped frame the review question, data extraction, reporting, and appraisal. The protocol of our systematic review has been previously registered at the PROSPERO International Prospective Register of Systematic Reviews website (under the reference CRD42022303197).

### Search strategy

We searched Medline, EMBASE, Cochrane, and Web of Science for review articles and regular research articles, from January 1st, 2011 to January 12th, 2022. Due to the lack of a unified definition for AKI prior to the introduction of KDIGO AKI criteria in 2012, we investigated studies published after 2011. Apart from restricting English language articles, no further restrictions were applied. Three search themes were used in the query: ”acute kidney injury”, ”outcome of AKI”, and ”artificial intelligence”. We also adapted these keywords to Medical Subject Heading (MeSH) terms according to the CHARMS guideline. To ensure consistency in the searches for all databases, first, we set up the search in Pubmed, then the query was translated to EMBASE, Cochrane, and Web of Science. A systematic search for grey literature was not carried out as it was deemed that searching across four databases would be sufficient. During the literature review of relevant studies, only one study was identified that did not surface through the search query. Table 1 shows our search strategy with every keyword and detail.

### Selection criteria

The purpose of this section is to discuss our criteria for including and excluding articles, and the steps taken by the reviewers to determine which articles were included or excluded.

#### Inclusion

Two independent reviewers (FNH and LV) screened all titles and abstracts identified by querying the databases using the search strategy detailed above. Articles identified as potentially relevant by either reviewer were subsequently read in full. Full-text articles were included if they (i) developed a machine learning-based or statistical prediction model for predicting renal insufficiency after an episode of AKI, and (ii) assessed the impact of the predictive model for renal insufficiency after an episode of AKI that was implemented in a clinical setting.

#### Exclusion

In this phase of the selection, articles were excluded based on the following criteria: (i) not a prediction model study, (ii) renal insufficiency is not the outcome, (iii) no validation of the model (neither internal nor external).

### Data extraction

The same two reviewers extracted data from the articles using a meticulously composed data extraction form that was designed in advance. The acquired data consists of: (i) the study setting, (ii) derivation and validation cohort descriptions, (iii) modeling approach, (iv) validation method, (v) model performance statistics, and (vi) final prediction tool design. We allowed details of external validation to be included in the extracted data when they were part of a preceding or sequential publication.

**Table 1:** Search strategy: keywords and MeSH terms for systematic literature review in Pubmed

Concept	Keywords *	MeSH terms
1. Acute Kidney Injury	”acute kidney injur*”, ”acute renal injur*, ”acute renal insufficienc*, ”acute kidney insufficienc*,“acute kidney failure*”, ”acute renal failure*”, ”renal insufficienc*”, ”kidney insufficienc*”, ”kidney dialys*”, ”renal dialys*”, ”hemodialys*”, ”hemodiafiltration”	”acute kidney injury”, ”renal insufficiency”
2. Outcome of AKI	”chronic renal insufficienc*”, ”chronic kidney insufficienc*”, ”chronic kidney disease*”, ”chronic renal disease*”, ”end-stage kidney disease*”, ”end-stage renal disease*”, ”end-stage kidney failure*”, ”chronic kidney failure”, ”chronic renal failure”, “ESRD”, ”follow-up stud*”, ”cohort stud*”, ”cohort analys*, ”follow-up”, ”long-term outcome*”	”renal insufficiency, chronic”, ”kidney failure, chronic”, ”follow-up studies”, ”cohort studies”,
3. AI/machine learning	”artificial intelligence”, ”machine intelligence”, ”computational intelligence”, ”statistical model*”, ”probabilistic model*”, ”decision support technique*”, ”decision support model*”, ”decision support system*”, ”decision analys*”, ”decision model”, ”predict model*”, ”prediction model*”, ”predict rule*”, ”predict score”, ”prediction score*”, ”prognostic model*”, ”decision rule”, ”risk model*”, ”risk algorithm*”, ”validation”, ”risk index”, ”risk predict*”, ”clinical model*” ”survival analysis”, ”proportional hazard model*”, ”Kaplan-Meier survival curve”, ”cox model*, ”time-to-event analysis”, ”machine learning”, ”transfer learning”, ”deep learning”, ”supervised machine learning”, ”learning from labeled data”, ”logistic model*”	”artificial intelligence”, ”models, statistical”, ”decision support techniques”, ”survival analysis”, ”risk”

* Throughout the table, * is truncation symbol.

Searches combined with AND: 1 AND 2 AND 3. The same search query has been adapted to be used in Web of Science, Cochrane, and Embase.

### Model performance

We gathered information concerning model discrimination and calibration using multiple units or by a combined measure, in order to evaluate the models’ performance. Calibration refers to the agreement between observed outcomes and predictions meaning that in this context if a model predicts a 40% risk of developing renal insufficiency for an AKI patient, the observed frequency of renal insufficiency should be approximately 40 out of 100 AKI patients with such a prediction [32]. The assessment of calibration consists of evaluating whether predicted probabilities and observed probabilities agree, including goodness-of-fit tests [for example, Hosmer–Lemeshow (HL) tests], table or graphical comparisons of predicted versus observed values within groups of predicted risks, or calibration plots. Poor calibration is indicated by an HL statistic with a small, significant p-value. Accordingly, discrimination is defined as the ability to distinguish between patients who are likely to develop renal insufficiencies such as acute kidney disease (AKD), which is a condition that falls between AKI and chronic kidney disease (CKD), and patients who are likely to develop CKD following an episode of AKI. Discriminating power was assessed using the area under the receiver operating characteristic (AUROC)/c-statistics [33]. Any information about the matching of model-predicted probabilities and observed probabilities was also included in the assessment of model performance, for example, the goodness-of-fit test, Hosmer-Lemeshow test [34], or table/graphical visualization of prediction versus observation values/performance.

### Study quality assessment

An assessment of quality criteria was conducted based on the Transparent Reporting of a multivariable prediction model for individual prognosis or diagnosis (TRIPOD statement) [35]. There is no standardized mechanism to assess the quality of impact analysis studies for risk prediction models. Therefore, quality criteria have been adapted from published articles that address the validity of prediction models in clinical implementation and impact analysis phases [36, 37].

## Results

### Characteristics of the included studies

We identified 33,746 potentially relevant abstracts from the searches over all of the databases. We also found one study from other sources and references. After the duplicate removal, as well as 25,812 title/abstract screening, 149 studies were assessed for full-text review. After full article screening, eight articles were identified for information extraction. As a result, we reviewed eight studies that reported prediction models.

**Fig. 2:**
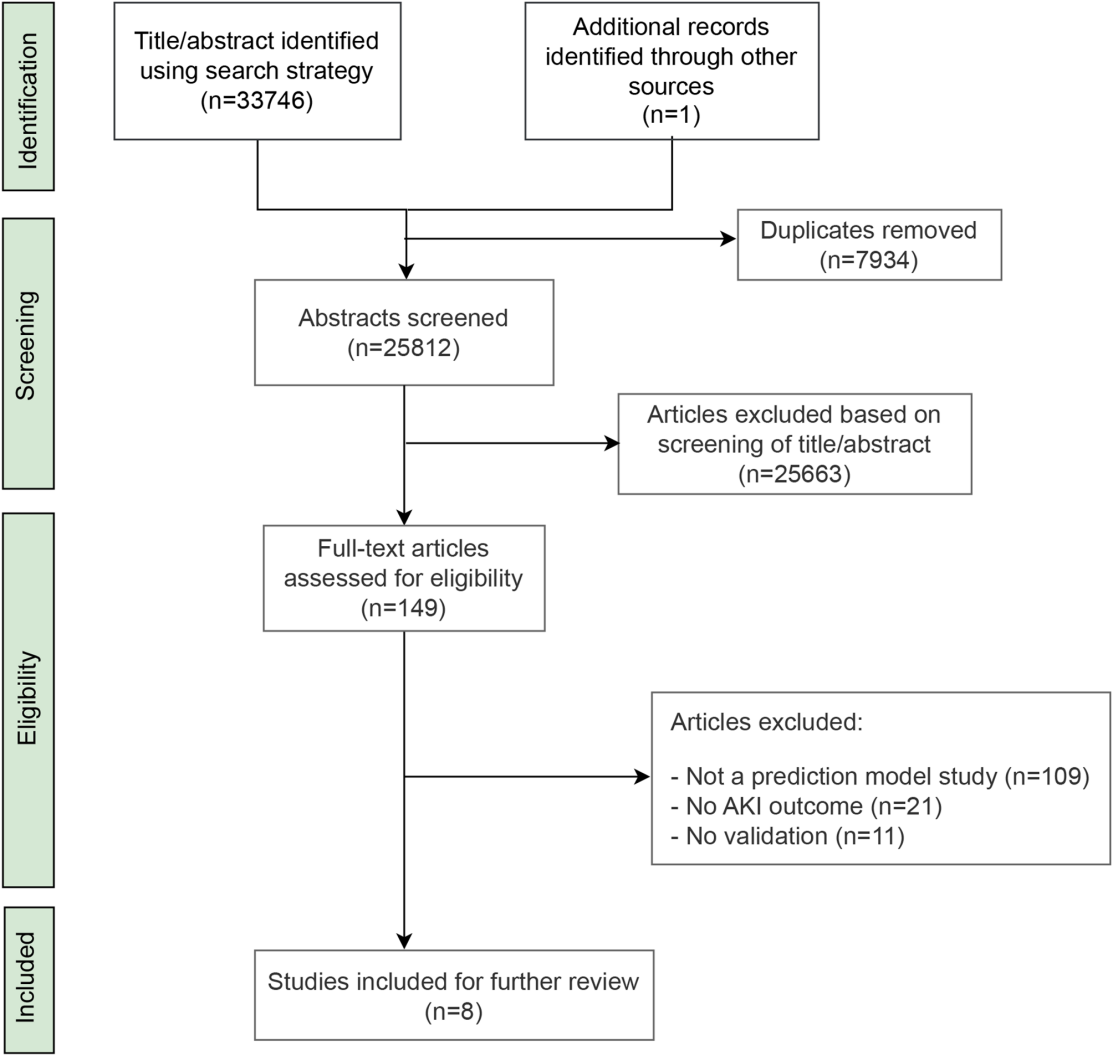
The flow of articles using our search strategy

Figure 2 shows the flow of articles based on our search strategy. A summary of the predictive variables included in the models is found in Table 2.

**Table 2:** Predictive variables included in the models. In the table, (✔) and (✘) indicate whether the variable has been used or not in the models, respectively

Variable	Chawla et al. [25]	Itenov et al. [38]	James et al. [39]	Lee et al. [40]
**Demographics**				
Age	✔	✔	✔	✔
Gender/Sex	Male/Female	Female	Male	✘
	African American/Hispanic/			
Race	Caucasian/Other	✘	✘	✘
**Laboratory data**				
Baseline serum creatinine, *mg/dL*	✘	✘	✔	✘
Serum creatinine, *mg/dL*	✔	✘	✘	✘
Discharge serum creatinine, *mg/dL*	✘	✘	✔	✘
Delta creatinine, *mg/dL*	✘	✔	✘	✘
Urinary output, *mL/kg/h*	✘	✔	✘	✘
Delta urinary output, *mL/kg/h*	✘	✘	✘	✘
Baseline eGFR, *mL/min/*1.73*m*^2^	✔	✘	✘	✘
Interleukin-8	✘	✘	✘	✘
Interleukin-16	✘	✘	✘	✘
AKI stage	✘	✘	1/2/3	✘
Albuminuria	✘	✘	Normal/Mild/Heavy/Unmeasured	✘
Baseline serum albumin (Alb)	✔	✘	✘	✘
Serum albumin (Alb)	✔	✘	✘	✘
Baseline serum hemoglobin (Hgb)	✔	✘	✘	✔
Serum hemoglobin (Hgb)	✔	✘	✘	✘
Total bilirubin	✘	✘	✘	✘
Maximum urea before the first AKI-3	✘	✘	✘	✘
Maximum white blood cell count before first AKI-3	✘	✘	✘	✘
Preadmission platelet count, *×*10^3^*/µl*	✘	✘	✘	✘
**Comorbidities**				
Apache II score	✘	✘	✘	✘
Oliguria	✘	✘	✘	✘
Mechanical ventilation	✘	✘	✘	✘
Diabetes mellitus (DM)	Yes/No	✘	✘	✘
	Never/During hospitalization/			
Dialysis	Post hospitalization	✘	✘	✘
Chronic liver disease	✘	✘	✘	Yes/No
Renal replacement therapy (RRT)	✔	✘	✘	✘
Arterial pH (Z-score)	✘	✘	✘	✘
Platelets	✘	✘	✘	✘
Mean arterial pressure	✘	✘	✘	✘
Acute tubular necrosis	Yes/No	✘	✘	✘
Time at risk (years)^1^	Yes/No	✘	✘	✘
Hospital complexity	1 A/1B/1 C/2/3	✘	✘	✘
Residency slots	✔	✘	✘	✘
Teaching hospital^2^	Yes/No	✘	✘	✘
Sepsis	✘	✘	✘	✘
Mechanical ventilation	✘	✘	✘	✘
Chronic obstructive pulmonary disease	✘	✘	✘	✘
APS III score	✘	✘	✘	✘
Diabetes	✘	✘	✘	✘
Congestive heart failure	✘	✘	✘	✘
Moderate or severe liver disease	✘	✘	✘	✘
SAPS II score	✘	✘	✘	✘
SOFA score	✘	✘	✘	✘
RRT on the first AKI-3 day in ICU	✘	✘	✘	✘
Hypertension	✘	✘	✘	✘
Surgery/trauma	✘	✘	✘	✘
Diuretic	✘	✘	✘	✘
Renal toxic drugs	✘	✘	✘	✘
Charlson Comorbidity Index	✘	✘	✘	✘
Emergency department	✘	✘	✘	✘
**Variable**	**Chen et al. [** 41 **]**	**He et al. [** 42 **]**	**Huang et al. [** 43 **]**	**Pike et al. [** 44 **]**
**Demographics**				
Age	✘	✔	✔	✔
Gender/Sex	✘	✔	✘	✘
BMI, *kg/m*^2^	✘	✔	✘	✘
**Laboratory data**				
Baseline serum creatinine, *mg/dL*	✘	✔	✘	✘
Serum creatinine, *mg/dL*	✘	✔	✘	✘
Delta creatinine, *mg/dL*	✔	✔	✘	✘
Urinary output, *mL/kg/h*	✘	✔	✘	✘
Delta urinary output, *mL/kg/h*	✘	✔	✘	✘
Baseline eGFR, *mL/min/*1.73*m*^2^	✘	✘	✘	✘
Interleukin-8	✔	✘	✘	✔
Interleukin-16	✔	✘	✘	✘
AKI stage	✘	1/2/3	✘	✘
Albuminuria	✘	✘	✘	✘
Baseline serum albumin (Alb)	✘	✘	✘	✘
Serum albumin (Alb)	✘	✘	✘	✘
Baseline serum hemoglobin (Hgb)	✘	✘	✘	✘
Serum hemoglobin (Hgb)	✘	✘	✘	✘
Total bilirubin	✘	✘	✘	✔
Maximum urea before first AKI-3	✘	✘	✔	✘
Maximum white blood cell count before first AKI-3	✘	✘	✔	✘
Preadmission platelet count, *×*10^3^*/µl*	✘	✘	✔	✘
**Comorbidities**				
Apache II score	✘	✘	✘	✔
Oliguria	✘	✘	✘	✔
Mechanical ventilation	✘	✘	✘	✔
Diabetes mellitus (DM)	✘	✘	✘	✘
Dialysis	✘	✘	✘	✘
Chronic liver disease	✘	✘	✘	✘
Renal replacement therapy (RRT)	✘	✘	✘	✘
Arterial pH (Z-score)	✘	✘	✘	✔
Platelets	✘	✘	✘	✔
Mean arterial pressure	✘	✘	✘	✔
Acute tubular necrosis	✘	✘	✘	✘
Time at risk (years)	✘	✘	✘	✘
Hospital complexity	✘	✘	✘	✘
Residency slots	✘	✘	✘	✘
Teaching hospital	✘	✘	✘	✘
Sepsis	✘	✘	Yes/No	✘
Mechanical ventilation	✘	✔	✘	✘
Chronic obstructive pulmonary disease	✘	✔	✘	✘
APS III score	✘	✔	✘	✘
Diabetes	✘	✔	✘	✘
Congestive heart failure	✘	Yes/No	✘	✘
Moderate or severe liver disease	✘	Yes/No	✘	✘
SAPS II score	✘	✔	✘	✘
SOFA score	✘	✔	✘	✘
RRT on the first AKI-3 day in ICU	✘	✘	✔	✘
Hypertension	✘	Yes/No	✘	✘
Surgery/trauma	✘	Yes/No	✔	✘
Diuretic	✘	Yes/No	✘	✘
Renal toxic drugs	✘	✔	✘	✘
Charlson Comorbidity Index	✘	✔	✘	✘
Emergency department	✘	✔	✘	✘
Renal toxic drugs	✘	✘	✘	✘
Charlson Comorbidity Index	✘	✘	✘	✘
Emergency department	✘	✘	✘	✘

^1^ Years from the diagnosis date to either the end of the data collection period or the date of death, whichever came first.

^2^ Teaching hospital was coded yes when the number of Medical Residents was ≥ 5.

### Summary of the included studies

Chawla et al. [25] conducted a prospective single-center cohort study in which they developed three prediction models to identify patients who survive AKI and are at higher risk for progression to stage 4 CKD. First, a model using all variables was developed, then a stepwise forward selection procedure with a threshold of P < 0.1 was used for feature selection. Then a second model was developed using the most heavily weighted factors from the first model. Following that, a third model was developed, called the ’bedside’ model, which is based on sentinel clinical events. Although model calibration was not reported for the study, in the model validation on the test set (separate validation cohort), models 1, 2, and 3 were all statistically significant in predicting progression to stage 4 CKD with c-statistics of 0.82, 0.81, and 0.77, respectively (P < 0.05 was the level of significance).

Itenov et al. [38] performed a multi-center prospective study on a cohort of adult critically ill patients admitted to the ICU for at least 24 hours and with AKI defined by KDIGO. The main outcome of this study was a recovery of kidney function within 28 days in which recovery is defined as living for five consecutive days with no renal replacement therapy and with creatinine levels below 1.5 times the baseline value (measured before ICU admission). The two developed models were validated on a separate validation cohort showing that 59.1% of the patients recovered, meaning that almost 40.9% of the patients developed any kind of renal insufficiency (e.g., different stages of CKD). In addition, 9.0% had a predicted chance of recovery of less than 25%, and their observed rate of recovery was 21.5%. The AUROC curve (or equivalently, the c-statistic) for predicting a recovery in the validation cohort was 73.1% (95% CI, 65.4–80.8%). Finally, calibration was described as nicely calibrated based on a graphical analysis of observed versus predicted probabilities.

James et al. [39] performed a multi-center prospective study in which they derived and internally as well as externally validated five different predictive models for the progression of AKI to advanced chronic kidney disease. Candidate predictor variables were selected based on previous studies. Then, stepwise backward variable selection with a significance level of P < 0.05 was used for the feature selection procedure. Five models with different variables were developed and out of all models, the first model (6-variable model) had the highest c-statistic of 0.87 (95% CI, 0.84–0.90) and 0.81 (95% CI, 0.75–0.86) in the internal and external validation cohort, respectively. Model calibration was described as well calibrated and was assessed by the calibration intercept, calibration slope, and graphically by locally weighted scatterplot smoothing (LOESS) plots of observed vs predicted probabilities of the outcome.

Lee et al. [40] published a multi-center retrospective cohort study on a cohort of dialysis-requiring adult acute kidney injury (AKI-D) patients who had predicted inpatient mortality of < 20%. The study aimed to develop and validate a prediction model for the probability of recovery in these patients. Different candidate predictors were used to develop two models using logistic regression and classification and regression tree (CART). Predicted recovery probabilities ranged from 9–22% in the lowest decile to 58–66% in the highest decile for logistic regression, and from 25.6–52.7% for the CART approach. The c-statistic was 0.64 and 0.61 for logistic regression and CART techniques, respectively. Based on a graphical comparison of observed probability to predicted probability, calibration was reported as excellent.

A separate study conducted by Chen et al. [41] analyzed 32 immunoinflammatory cytokines in the blood of patients with cardiac surgery-associated acute kidney injury (CSA-AKI) and then employed machine learning methods to develop a simple and effective blood marker-based model for predicting poor in-hospital outcomes. CSA-AKI, defined as abrupt renal dysfunction that occurs in patients following cardiac surgery, is a prevalent complication affecting approximately 5 percent to 42 percent of patients undergoing cardiac surgery [45]. Using both the Least Absolute Shrinkage and Selection Operator (LASSO) and random forest predictor selection methods, they showed a logistic regression-based predictive model incorporating IL-8, IL-16, and a change in SCr assists in accurately predicting poor in-hospital outcomes. Their prediction model was effective at predicting composite outcomes, reporting AUROC of 0.947 (95% CI, 0.895–0.998) and 0.971 (95% CI, 0.932-1.000) for internal and external validation, respectively. Model calibration was assessed by Brier score and Hosmer–Lemeshow test for external validation and reported as good calibration (Brier score 0.094, HL test P value = 0.103).

In a separate study that studied the outcome in critically ill patients with sepsis-associated AKI, He et al. (2021) [42] developed and validated machine learning models to predict the occurrence of AKD. AKD was defined as the presentation of at least KDIGO Stage 1 criteria for > 7 days after an AKI-initiating event [46]. To determine the most useful predictive variables, LASSO has been used and 28 variables (listed in Table 2) have been selected for inclusion in the predictive models. The results of three different models, including recurrent neural network-long short-term memory (RNN-LSTM), decision tree, and logistic regression, were compared on two separate training and validation (MIMIC III) datasets. In the validation dataset, the RNN-LSTM algorithm showed the highest performance with an AUROC of 1.000, followed by the decision trees with an AUROC of 0.872. Logistic regression had the least predictive accuracy, with an AUROC of 0.717. The calibration curve was provided and reported as being well-calibrated.

Recently, Huang et al. [47] developed and validated prediction models for AKI recovery in critically ill patients at hospital discharge with ICU-acquired AKI stage 3 (AKI-3). After internal (10-fold cross-validation) and external validation the prediction LASSO model for complete or partial recovery based on age, need for RRT, platelet count, urea, and white blood cell count had the highest AUROC of 0.61. Moreover, calibration was evaluated visually with a calibration slope of 0.27 and 0.32, and calibration in the large of -0.07 and zero for complete recovery prediction and complete or partial recovery prediction models, respectively. Models that are well calibrated will have calibration plots close to the diagonal axis, a calibration slope close to one, and a calibration in the large close to zero.

Finally, Pike et al. [44], reported a multi-center prospective cohort study aiming to develop a biomarker-enhanced risk pre- diction model for critically ill patients receiving RRT with AKI. They investigate whether plasma inflammatory and apoptosis biomarkers increase risk prediction of renal recovery and mortality compared with clinical models in which the primary outcomes of interest were renal recovery and mortality at day 60. Four different models were developed using multivariate logistic regression in which each model uses a different set of variables (see Table 3). The c-statistic for all biomarkers for recovery and mortality were 0.66 and 0.71, respectively. The results show that a simple four-variable clinical model including age, mean arterial pressure, mechanical ventilation, and bilirubin, together with IL-8, increases prediction quality (AUROC, 0.76; 95% CI, 0.71–0.81) for renal recovery at day 60 and could potentially be beneficial at the bedside for clinicians. Calibration performance was assessed using the Hosmer–Lemeshow (HL) goodness-of-fit test and reported as good calibration (P value range, 0.08–0.45).

**Table 3:** AKI-outcome prediction models

	Chawla et al. [25]	Itenov et al. [38]	James et al. [39]	Lee et al. [40]
**Model development**				
Sample of patients	Patients who survive AKI	Patients admitted to the ICU for at least 24 h and with AKI	patients with a prehospitalization eGFR of more than 45 mL/min/1.73m^2^ and who had survived hospitalization with AKI	Adult (age > 18 years) who developed dialysis-requiring AKI (AKI-D)
Study design	Prospective cohort study	Prospective cohort study	Prospective cohort study	Retrospective cohort study
Number of centers	1 center	9 academic ICUs	Multicenter (population-based repository)	21 hospitals
AKI definition	RIFLE	KDIGO	KDIGO	RRT + SCr > 50% rise
Derivation cohort sample size	5351	568	9973	2214
Derivation time period	October 1999 - December 2005	2006–2010	April 2004 - March 2014, with follow-up to March 2015	January 2009 - September 2015
The outcome of interest	Risk for progression to CKD stage 4	Recovery after AKI within 28 days	Progression of AKI to advanced CKD	Recovery after dialysis-requiring AKI within 90 days
Number of prediction models	Three logistic regression models	Two cause-specific Cox regression models: one for the hazard of recovery and one for death without recovery	Five multivariate logistic regression	Two models: Logistic regression and classification and regression tree (CART)
Predictor selection method (e.g.full model approach, backward elimination)	Model1: stepwise logistic regression, Model2: based on the most heavily weighted factors from model1, Model3: based on sentinel clinical events	Model1: most likely predictors, Model2: full model	Stepwise backward logistic regression at P < 0.05 with bootstrap selection (1000 samples)	Stepwise logistic regression with bootstrap selection (1000 samples)
Incidence of outcome	13.6% entered CKD4	15.1% risk of not recovering	2.7% developed advanced CKD	59.1% not recovered after AKI-D
Validation method				
Validation cohort sample (e.g. split sample, bootstrap)	Separate cohort	Separate cohort	Internal (one-third of derivation cohort) and separate cohort	Internal validation (10-fold cross-validation)
Validation cohort sample size	11,589	766	2761 (external cohort)	-
Validation time period	October 1999 - December 2005	1 January 2012–31 December 2013	June 2004 - March 2012, with a follow-up to March 2013	January 2009 - September 2015
Incidence of outcome	8.5% entered CKD4	10% risk of not recovering	2.2% developed advanced CKD	59.1% not recovered after AKI-D
Performance statistics	c − statistics = 0.81–0.82	AUROC = 73.1% for predicting recovery	c − statistic = 0.87	Logistic regression: c − index = 0.645, CART: c − index = 0.61
Model performance statistics: calibration	Not reported	The calibration plot used, noted as nicely calibrated	P (slope) = 0.92, 0.88, 0.8, 0.89, 0.67	The calibration plot used, noted as excellent calibration
	**Chen et al. [** 41 **]**	**He et al. [** 42 **]**	**Pike et al. [** 44 **]**	**Huang et al. [** 43 **]**
**Model development**				
Sample of patients	Patients diagnosed with cardiac surgery-associated AKI (CSA-AKI)	Patients with sepsis-associated AKI	Critically ill patients receiving RRT with AKI	ICU patients with AKI-3
Study design	Prospective cohort study	Prospective cohort study	Prospective cohort study	Prospective cohort study
Number of centers	1 center	1 center	Multicenter	Multicenter (seven ICUs)
AKI definition	Not mentioned	KDIGO	Not mentioned	KDIGO
Derivation cohort sample size	196	209	1124	229
Derivation time period	not mentioned	January 2015 - December 2020	November 2003 - July 2007	August 2007 - November 2010
The outcome of interest	Postoperative AKI requiring RRT or in-hospital death	Predict the occurrence of acute kidney disease (AKD) in patients with sepsis-associated AKI	Renal recovery and mortality for ill patients with AKI requiring RRT at day 60	Two outcomes: 1) complete recovery and 2) complete or partial recovery at hospital discharge
Number of prediction models	Five logistic regression models with different combinations of the 3 selected predictors	Three models: Recurrent Neural Network-Long Short-Term Memory (RNN-LSTM), decision trees, and logistic regression	Four logistic regression models (ATN clinical model, reduced ATN model, LASSO model, stepwise-selected model, and parsimonious model)	Multiple Least absolute shrinkage and selection operator (LASSO) models
Predictor selection method (e.g. full model approach, backward elimination)	LASSO logistic regression and random forests	LASSO	Model1: reduced ATN model, Model2: LASSO, Model3: stepwise logistic regression, Model4: routinely available predictors	Correlation-based feature selection (n = 4) and one feature added based on the literature
Incidence of outcome	16.3%	55.5%	36.5%	37.55% (complete recovery)
Validation method				
Validation cohort sample (e.g. split sample, bootstrap)	Internal validation (bootstrap) and separate cohort	Separate cohort (MIMIC III database)	Internal validation (2-fold split)	Internal validation (stratified 10-fold cross-validation) and a separate cohort
Validation cohort sample size	52	509	562	244
Validation time period	Not mentioned	2008–2014	November 2003 - July 2007	August 2007 - November 2010
Incidence of outcome	21.1%	46.4%	-	33.20% (complete recovery)
Performance statistics	ROC-AUC = 97.1%	AUROC for LSTM = 1.00 AUROC for decision trees = 0.872 AUROC for logistic regression = 0.717	Renal recovery using model 4: AUROC = 0.76%	Complete recovery: AUROC = 0.53%, complete or partial recovery: AUROC = 0.61%
Model performance statistics: calibration	Calibration score assessed by Brier score and HL test and noted as good	The calibration plot used, noted as nicely calibrated	HL: P = 0.08–0.45	Calibration plot used

**Fig. 3:**
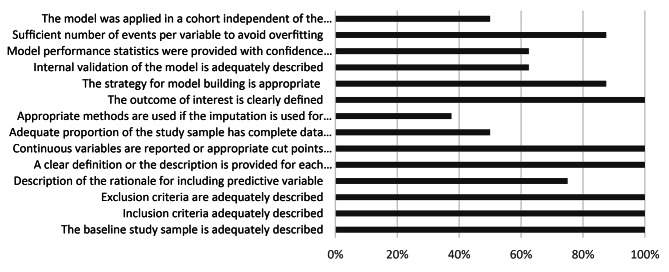
Percentage of studies meeting quality criteria

A comparative summary of all clinical prediction models is shown in Table 3 and a summary of their methodological quality is provided in Fig. 3.

### Quality assessment summary

Table 4 shows the quality assessment of model development of the included studies. “As a whole, the quality measures reflected by the studies are rather average or below average, for example, only 40% of quality criteria are met by all the studies. All studies except the ones by Chawla et al. [25] and He et al. [42] described the rationale for including predictive variables. However, only three studies by Chawla et al. [25], Huang et al. [43], and Pike et al. [44] discussed handling missing data. The number of events per variable was < 10 for the study conducted by He et al. [42], and four of the eight models were validated externally.

**Table 4:** Quality assessment of model development

	Chawla et al. [25]	Itenov et al. [38]	James et al. [39]	Lee et al. [40]	Chen et al. [41]	He et al. [42]	Huang et al. [43]	Pike et al. [44]
The baseline study sample is adequately described for key characteristics	Y	Y	Y	Y	Y	Y	Y	Y
Inclusion criteria adequately described	Y	Y	Y	Y	Y	Y	Y	Y
Exclusion criteria are adequately described	Y	Y	Y	Y	Y	Y	Y	Y
Description of the rationale for including predictive variable	N	Y	Y	Y	Y	N	Y	Y
A clear definition or the description is provided for each predictive variable	Y	Y	Y	Y	Y	Y	Y	Y
Continuous variables are reported or appropriate (i.e. not data-dependent) cut points are used	Y	Y	N- goodness-of-fitness tests used to maximize model fit	Y	Y	Y	Standardized to zero, mean, and unit variance	Y
Adequate proportion of the study sample has complete data for prognostic factors	Y	Not reported	Not reported	Y	Not reported	Not reported	Y	Y
Appropriate methods are used if the imputation is used for missing prognostic factor data	Complete case analysis	Not reported	Not reported	Not reported	Not reported	Not reported	Mean for continuous data and the mode for categorical	Multiple imputations
The outcome of interest is clearly defined	Y	Y	Y	Y	Y	Y	Y	Y
The strategy for model building (i.e. inclusion of variables) is appropriate and is based on a conceptual framework or model (i.e. adequate description of mathematical techniques to derive the model)	Y—stepwise multivariate	Y—full model	Y—backward selection	Y—stepwise multivariate	Y—LASSO and random forests	Y—LASSO	Y—correlation-based	Y—LASSO and stepwise multivariate
Internal validation of the model is adequately described (e.g. bootstrapping, cross-validation, or internal validation cohort details are provided)	Not reported	Not reported	Y	Y	Y	Not reported	Y	Y
Model performance statistics were provided with confidence intervals (e.g. ROC curves/c-statistic, HL statistics, likelihood ratios, PPV or NPV)	Y	Y	Y	Y (point estimate only)	Y	Y (point estimate only)	Y (point estimate only)	Y
A sufficient number of events per variable to avoid overfitting (e.g. *>*10)	Y	Y	Y	Y	Y	N	Y	Y
The model was applied in a cohort independent of the development cohort and the model’s predictive performance was assessed	Y	Y	N	N	N	Y	N	Y

## Discussion

In this systematic review, we aimed to find prediction models for the development of renal insufficiency (or recoveries) in patients who experienced AKI. We identified eight studies in which multiple prediction models were built and validated in heterogeneous cohorts of patients. The quality of the studies and the models developed are rather average in general.

AKI was defined using the KDIGO criteria in four studies [38, 39, 42, 48], and one study used the RIFLE criteria [25], the other three studies did not mention the used AKI criteria [40, 41, 44]. Our systematic review found some limitations in the derivation and validation of all published studies. For a model to be generalizable beyond a sample population, validation is an essential step. Although all the models underwent some internal validation and reported model calibration (except Chawla et al. [25]), not all of them were externally validated. In addition, internal validation in one of the studies was performed in a random split of the dataset [44], which is not a perfect method for data splitting in that it generates quite similar development and validation set. While some studies did not mention how missing values were handled, of those that did, the majority relied on relatively simple methods, such as complete case analysis and single imputation using mean for continuous data and the mode for categorical data. Only one study used a regression-based algorithm [44]. Multiple imputation methods have proven to be more effective than single imputation methods at restoring the natural variability of missing values and retaining more useful information than complete case analysis methods [49].

Moreover, three of the studies selected risk factors using LASSO for variable selection [41, 42, 44]. However, four of the eight models used statistical approaches of forward selection or backward elimination [25, 39, 40, 44], and one used correlation-based techniques [48]. Studies conducted using stepwise regression techniques have demonstrated wide variation in models selected from a list of candidate predictors. By bootstrapping for predictor selection, model developers can take into account this variability since the final candidate predictors are those selected by a predetermined majority of bootstrap samples. Only one model was developed using a full model approach. In addition, three of the studies only focused on one particular center [25, 41, 42]. Using a single-center cohort may not be representative of other populations. While half of the models had only average to poor predictive power with AUROC values below 80%, good results were obtained in studies involving selected cohorts (cardiac surgery, sepsis). In addition, the majority of the studies used small derived and validated cohorts, and in all studies, all models were validated in cohorts from the same region, so generalizability to patients from other regions was not examined. Moreover, all studies excluded patients with preexisting CKD, therefore these prediction models may not be accurate in that population. In the included studies, conventional statistical models or simple machine learning techniques such as CART, RNN, and logistic regression were the methods employed in this area. Rajula et al. [50] showed the traditional statistical method seems more useful than machine learning models when the number of cases is greater than the number of variables when applied to the medical field. However, in scenarios where the number of variables is large, traditional statistical models might run into problems. EHRs are capable of storing a large number and variety of variables enabling high-quality and trustworthy prediction models [51], and machine learning offers the techniques to handle large amounts of high-dimensional data where the number of variables is huge which is common in healthcare settings. Besides, these machine learning models are capable of capturing complex interactions between the variables in the datasets, resulting in more precise and reliable models. However, statistical models that leverage the diversity and abundance of EHR-derived data are still limited. Furthermore, many machine learning models like random forest [52] are able to handle missing values (one of the main challenges when developing EHR-based models) naturally, without the need to include a data imputation step. Also, the interpretability of model predictions is an important consideration when implementing and utilizing them by clinical providers and other healthcare decision-makers, and some machine learning models such as decision trees and random forests can be more easily interpreted. Despite many advantages, most machine learning models (e.g., deep learning) are computationally expensive and need more time for training. Despite the fact that hyperparameter selection can greatly influence the performance of a model, hyperparameter selection is often neglected in these studies [53]. It is our understanding that there are no guidelines regarding how to report the hyperparameter tuning results/procedure for machine learning as clinical prediction models. Another important issue is the limited amount of follow-up data. Based on the results of included papers, the need for early detection and prevention of AKI is important. However, currently, after discharge from the hospital, the follow-up of AKI survivors is considerably challenging mainly due to two reasons. First, the process is time-consuming and costly, and second, drop-out is frequently observed [54]. As a result, when developing machine learning-based CKD risk prediction models for such patients, we are typically confronted with a small, labeled training set. For future research, we propose organizing longer follow-up studies of AKI patients, utilizing advanced machine learning methods to take into account as many variables as possible, and employing techniques of semi-supervised learning to deal with probable dropouts [55].

It is important to note that this systematic review has both strengths and limitations. This is the first systematic review to examine both the reporting quality and the development of machine learning models that predict outcomes of AKI. Although we used standard search filters for AKI, outcomes of AKI, and machine learning, we may not have found all relevant studies in the databases that we have looked into or studies that are not included in these databases and not published in English, resulting in only 8 studies included in the systematic review. In addition, although all studies provide prediction models to predict renal insufficiency outcomes in AKI patients, heterogeneous outcomes (progression to CKD, progression to AKD, renal recovery, and requiring RRT) are provided in these studies. Moreover, it was not possible to perform a meta-analysis of the studies because access to individual participant data was not available. Finally, an individual model cannot be recommended or implemented due to the limited number of externally validated models and the absence of an impact analysis.

## Conclusion

In recent years, few validated clinical models have been developed that can predict the outcomes of acute kidney injury in critically ill or hospitalized patients. The existence and use of such models, in addition to highlighting increased renal insufficiency, morbidity, and mortality following AKI, have significant implications for the future care needs of survivors. Future studies using machine learning prediction algorithms may improve the model design that can be better used in the clinical setting.

## Data Availability

The datasets used and/or analyzed during the current study are available from the corresponding author upon reasonable request.

## References

[1] Bellomo R, Kellum JA, Ronco C. “Acute kidney injury,” *Lancet*, vol. 380, no. 9843, pp. 756–766, Aug. 2012. 10.1016/S0140-6736(11)61454-2 22617274

[2] Wang HE, Muntner P, Chertow GM, Warnock DG. Acute kidney Injury and Mortality in Hospitalized Patients. Am J Nephrol. May 2012;35(4):349–55. 10.1159/000337487 PMC3362180 22473149

[3] Case J, Khan S, Khalid R, Khan A. “Epidemiology of acute kidney injury in the intensive care unit,” *Crit. Care Res. Pract*, vol. 2013, 2013. 10.1155/2013/479730 PMC3618922 23573420

[4] Hoste EAJ (2018). Global epidemiology and outcomes of acute kidney injury. Nat Rev Nephrol.

[5] Bagshaw SM, George C, Bellomo R, Committee ADM. and others, “Early acute kidney injury and sepsis: a multicentre evaluation,” *Crit. Care*, vol. 12, no. 2, p. R47, 2008. 10.1186/cc6863 PMC2447598 18402655

[6] Kellum JA (2012). Kidney disease: improving global outcomes (KDIGO) acute kidney injury work group. KDIGO clinical practice guideline for acute kidney injury. Kidney Int Suppl.

[7] Sparrow HG, Swan JT, Moore LW, Gaber AO, Suki WN (2019). Disparate outcomes observed within kidney disease: improving global outcomes (KDIGO) acute kidney injury stage 1. Kidney Int.

[8] Nateghi Haredasht F, Antonatou M, Cavalier E, Delanaye P, Pottel H, Makris K. “The effect of different consensus definitions on diagnosing acute kidney injury events and their association with in-hospital mortality,” *J. Nephrol*, vol. 35, no. 8, pp. 2087–2095, Nov. 2022. 10.1007/s40620-022-01323-y 35441981

[9] Dharnidharka VR, Kwon C, Stevens G (2002). Serum cystatin C is superior to serum creatinine as a marker of kidney function: a meta-analysis. Am J Kidney Dis.

[10] Helmersson-Karlqvist J et al. “Cystatin C predicts long term mortality better than creatinine in a nationwide study of intensive care patients,” *Sci. Reports 2021 111*, vol. 11, no. 1, pp. 1–9, Mar. 2021. 10.1038/s41598-021-85370-8 PMC7961058 33723337

[11] Nateghi Haredasht F, Viaene L, Vens C, Callewaert N, De Corte W, Pottel H. “Comparison between Cystatin C- and Creatinine-Based Estimated Glomerular Filtration Rate in the Follow-Up of Patients Recovering from a Stage-3 AKI in ICU,” *J. Clin. Med*, vol. 11, no. 24, Dec. 2022. 10.3390/jcm11247264 PMC9784749 36555881

[12] Clerico A, Galli C, Fortunato A, Ronco C. “Neutrophil gelatinase-associated lipocalin (NGAL) as biomarker of acute kidney injury: A review of the laboratory characteristics and clinical evidences,” *Clin. Chem. Lab. Med*, vol. 50, no. 9, pp. 1505–1517, Sep. 2012. 10.1515/cclm-2011-0814 22962216

[13] Devarajan P. “NGAL in Acute Kidney Injury: From Serendipity to Utility,” *Am. J. Kidney Dis*, vol. 52, no. 3, pp. 395–399, Sep. 2008. 10.1053/j.ajkd.2008.07.008 18725011

[14] Soni SS et al. “NGAL: A biomarker of acute kidney injury and other systemic conditions,” *Int. Urol. Nephrol*, vol. 42, no. 1, pp. 141–150, Mar. 2010. 10.1007/s11255-009-9608-z 19582588

[15] Srisawat N (2011). Urinary biomarkers and renal recovery in critically ill patients with renal support. Clin J Am Soc Nephrol.

[16] Murugan R (2010). Acute kidney injury in non-severe pneumonia is associated with an increased immune response and lower survival. Kidney Int.

[17] Sileanu FE (2015). AKI in low-risk versus high-risk patients in intensive care. Clin J Am Soc Nephrol.

[18] Network VARFT (2008). Intensity of renal support in critically ill patients with acute kidney injury. N Engl J Med.

[19] Beker BM, Corleto MG, Fieiras C, Musso CG (2018). Novel acute kidney injury biomarkers: their characteristics, utility and concerns. Int Urol Nephrol.

[20] Hashemian SM (2016). Outcome of acute kidney injury in critical care unit, based on AKI network. Tanaffos.

[21] Fuchs L (2013). Severity of acute kidney injury and two-year outcomes in critically ill patients. Chest.

[22] Levey AS (2007). Chronic kidney disease as a global public health problem: approaches and initiatives–a position statement from kidney disease improving global outcomes. Kidney Int.

[23] Hsu RK, Hsu C. “The role of acute kidney injury in chronic kidney disease,” in *Seminars in nephrology*, 2016, vol. 36, no. 4, pp. 283–292. 10.1016/j.semnephrol.2016.05.005 PMC4979984 27475659

[24] Silver SA et al. “Ambulatory care after acute kidney injury: an opportunity to improve patient outcomes,” *Can. J. kidney Heal. Dis*, vol. 2, no. 1, Oct. 2015. 10.1186/s40697-015-0071-8 PMC4595050 26445676

[25] Chawla LS, Amdur RL, Amodeo S, Kimmel PL, Palant CE (2011). The severity of acute kidney injury predicts progression to chronic kidney disease. Kidney Int.

[26] Silver SA et al. “Nephrologist follow-up versus usual care after an acute kidney injury hospitalization (Fusion): A randomized controlled trial,” *Clin. J. Am. Soc. Nephrol*, vol. 16, no. 7, pp. 1005–1014, Jul. 2021. 10.2215/CJN.17331120 PMC8425610 34021031

[27] Kellum JA, Prowle JR (2018). Paradigms of acute kidney injury in the intensive care setting. Nat Rev Nephrol.

[28] Boonstra A, Versluis A, Vos JFJ (2014). Implementing electronic health records in hospitals: a systematic literature review. BMC Health Serv Res.

[29] Panahiazar M, Taslimitehrani V, Pereira N, Pathak J (2015). Using EHRs and machine learning for heart failure survival analysis. Stud Health Technol Inform.

[30] Kawaler E, Cobian A, Peissig P, Cross D, Yale S, Craven M. “Learning to predict post-hospitalization VTE risk from EHR data,” in *AMIA annual symposium proceedings*, 2012, vol. 2012, p. 436. PMC3540493 23304314

[31] Callahan A, Shah NH. “Machine learning in healthcare,” in Key Advances in Clinical Informatics,Elsevier, 2017,pp. 279–291.

[32] Hilden J, Habbema JDF, Bjerregaard B (1978). The measurement of performance in probabilistic diagnosis. Methods Inf Med.

[33] Hanley JA, McNeil BJ (1982). The meaning and use of the area under a receiver operating characteristic (ROC) curve. ” Radiol.

[34] Hosmer DW. “Assessing the fit of the model,”Appl. Logist. Regres., pp.143–202, 2000.

[35] Collins GS, Reitsma JB, Altman DG, Moons KGM (2015). Transparent reporting of a multivariable prediction model for individual prognosis or diagnosis (TRIPOD) the TRIPOD Statement. Circulation.

[36] Hayden JA, Côté P, Bombardier C. “Evaluation of the quality of prognosis studies in systematic reviews,” *Ann. Intern. Med*, vol. 144, no. 6, pp. 427–437, Mar. 2006. 10.7326/0003-4819-144-6-200603210-00010 16549855

[37] Wilson T (2016). Risk prediction models for acute kidney injury following major noncardiac surgery: systematic review. Nephrol Dial Transplant.

[38] Itenov TS (2018). Predicting recovery from acute kidney injury in critically ill patients: development and validation of a prediction model. Crit Care Resusc.

[39] James MT (2017). Derivation and external validation of prediction models for advanced chronic kidney disease following acute kidney injury. JAMA.

[40] Lee BJ (2019). Predicting renal recovery after dialysis-requiring acute kidney injury. Kidney Int reports.

[41] Chen Z et al. “A novel predictive model for poor in-hospital outcomes in patients with acute kidney injury after cardiac surgery,” *J. Thorac. Cardiovasc. Surg*, May 2021. 10.1016/j.jtcvs.2021.04.085 34112503

[42] He J, Lin J, Duan M. “Application of Machine Learning to Predict Acute Kidney Disease in Patients With Sepsis Associated Acute Kidney Injury,” *Front. Med*, vol. 0, p. 2407, Dec. 2021. 10.3389/fmed.2021.792974 PMC8703139 34957162

[43] Huang CY et al. “Development and validation of clinical prediction models for acute kidney injury recovery at hospital discharge in critically ill adults,”J. Clin. Monit. Comput., 2022. 10.1007/s10877-022-00865-7 35532860

[44] Pike F (2015). Biomarker enhanced risk prediction for adverse outcomes in critically ill patients receiving RRT. Clin J Am Soc Nephrol.

[45] Hobson CE, et al. Acute kidney injury is associated with increased long-term mortality after cardiothoracic surgery. Circulation. May 2009;119(18):2444–53. 10.1161/CIRCULATIONAHA.108.800011 19398670

[46] Chawla LS, et al. Acute kidney disease and renal recovery: consensus report of the Acute Disease Quality Initiative (ADQI) 16 workgroup. Nat Rev Nephrol. Feb. 2017;2017 134(4):241–57. 10.1038/nrneph.2017.2 28239173

[47] Huang CY et al. “Development and validation of clinical prediction models for acute kidney injury recovery at hospital discharge in critically ill adults,”J. Clin. Monit. Comput., pp.1–13, May 2022. 10.1007/s10877-022-00865-7 35532860

[48] Huang C-Y et al. “Development and validation of clinical prediction models for acute kidney injury recovery at hospital discharge in critically ill adults.&#8221 10.1007/s10877-022-00865-7 35532860

[49] Sterne JAC et al. “Multiple imputation for missing data in epidemiological and clinical research: potential and pitfalls,” *BMJ*, vol. 338, no. 7713, pp. 157–160, Jul. 2009. 10.1136/bmj.b2393 PMC2714692 19564179

[50] Rajula HSR, Verlato G, Manchia M, Antonucci N, Fanos V. “Comparison of Conventional Statistical Methods with Machine Learning in Medicine: Diagnosis, Drug Development, and Treatment,” *Med. 2020, Vol. 56, Page 455*, vol. 56, no. 9, p. 455, Sep. 2020. 10.3390/medicina56090455 PMC7560135 32911665

[51] Kennedy EH, Wiitala WL, Hayward RA, Sussman JB (2013). Improved cardiovascular risk prediction using nonparametric regression and electronic health record data. Med Care.

[52] Breiman L (2001). Random forests. Mach Learn.

[53] Luo G. A review of automatic selection methods for machine learning algorithms and hyper-parameter values. Netw Model Anal Heal Informatics Bioinforma. Dec. 2016;5(1):1–16.

[54] McDonald HI, Shaw C, Thomas SL, Mansfield KE, Tomlinson LA, Nitsch D (2016). Methodological challenges when carrying out research on CKD and AKI using routine electronic health records. Kidney Int.

[55] Nateghi Haredasht F, Vens C. “Predicting Survival Outcomes in the Presence of Unlabeled Data,” *Mach. Learn*, Nov. 2022.

